# Psychometric Properties of the English and Malay Version of the Adapted Schutte Emotional Intelligence Scale

**DOI:** 10.3389/fpsyg.2022.895816

**Published:** 2022-10-10

**Authors:** Nor Aniza Ahmad, Sarva Mangala Praveena, Ker Shin Tee

**Affiliations:** ^1^Department of Foundations of Education, Faculty of Educational Studies, Universiti Putra Malaysia, Serdang, Malaysia; ^2^Department of Environmental and Occupational Health, Faculty of Medicine and Health Sciences, Universiti Putra Malaysia, Serdang, Malaysia

**Keywords:** perception of emotions, utilization of emotion, understand emotion, managing own emotions, managing others' emotions, emotional intelligence, psychometric

## Abstract

The study examines the psychometric properties of the adapted Schutte Emotional Intelligence Scale (A-SEIS) with 200 undergraduate students at the Universiti Putra Malaysia (UPM). Upon the permission, the researchers adapted the original instrument, SEIS by incorporating a new construct which is understanding of emotions and some ability-based items into the perceived emotions construct. The A-SEIS is a mixed (trait and ability) measure EI instrument that aims at assessing four important dimensions of EI, including perception of emotions, utilization of emotions, understanding of emotions, and management of emotions. The study investigated the content validity of the A-SEIS by using the content validity indexing (CVI). Three expert panels translated and back-translated the A-SEIS and rated the degree of relevance of every item based on the four-point scale provided in the content validation form. The exploratory factor analysis (EFA) methods were used to explore the underlying structure of the A-SEIS. The general validity testing of the adapted instrument was carried out in the framework of the structural equation modeling (SEM) approach by applying two iterations of confirmatory factor analysis (CFA), the first approach is the covariance-based SEM (CB-SEM) approach, followed by the partial least squares based SEM (PLS-SEM) using two different software: AMOS and smartPLS. Research findings concluded that the instrument is reliable and valid to be applied in tertiary education settings and future research.

## Introduction

Emotional intelligence (EI) is one of the most widely discussed topics of intelligence in the current literature. With its population, many EI instruments have been developed to measure and predict the EI skills, for example, the Mayer–Salovey–Caruso Emotional Intelligence Test (MSCEIT), the Schutte Emotional Intelligence Scale (SEIS), the Emotional Competence Inventory (ECI), the Bar-On Emotional Quotient Inventory (EQ-i), the Wong and Law Emotional Intelligence Scale, among others. Among these EI instruments, the SEIS is one of the tests that has been widely adopted to measure individuals' EI abilities (Grant, 2007; Hen and Sharabi-Nov, 2014). Originally, the SEIS was developed using the Salovey and Mayer's (1990) EI model and the scale scores can be used to predict persistence, adjustment of fresh graduates to university, mood repair, and academic grades (as cited in Schutte et al., 2009). SEIS is an English version of a self-report inventory containing 33 items with four dimensions: perception of emotions, managing own emotions, managing others' emotions, and utilization of emotions. This instrument was developed by Schutte et al. (2009) with Cronbach's alpha (CA) of 0.87 and test–retest reliability of 0.78. The predicted validity for first-year grade point average (GPA) of college students is *r*(63) = 0.32, the discriminant validity for the correlation between the SEIS and Scholastic Assessment Test (SAT) scores is *r*(41) = −0.06, and the subscales of revised NEO Personality Inventory score of college students is *r*(22) = −0.21 to 0.54 (Schutte et al., 1998). The current study adapted the SEIS based on Salovey and Mayer's (1990) newer EI model. In a nutshell, the researchers attempted to investigate three main research questions (RQ): RQ1: What are the content validity index (CVI) and kappa statistic coefficient of the adapted Schutte Emotional Intelligence Scale (A-SEIS)? RQ2: What are the reliability and validity of the adapted Schutte Emotional Intelligence Scale (A-SEIS)? RQ3: Are there any associations among various factors in the adapted instrument?

### Ability, Trait, and Mixed Measure of EI

SEIS is a self-report inventory that focuses on measuring the typical or trait EI. Salovey and Mayer's (1990) EI model defined EI as a combination of what might be considered an ability and trait and the latest model argued for pure ability. The SEIS measures the constructs of Salovey and Mayer's (1990) model of EI: perception of emotions, managing emotions, and utilization of emotions. As declared, Salovey and Mayer (1990) had refined their ability-based model from 3 constructs into four-branch model of EI abilities. Although some changes have been made, yet the basic concepts of newer model are about the same as the older model (as cited in Schutte et al., 2009). Thus, the later added construct, understanding of emotion, was not incorporated into the instrument.

Mayer et al. (2008) have declared that the four-branch model of EI is most promising for future development. The latest ability model defined by Mayer and Salovey (1997) consisted of perceiving one's own and others' emotions, utilization of emotions, understanding of emotions, and managing one's own and others' emotions (as cited in Warwick et al., 2010). They developed the MSCEIT to measure the latest ability-based EI model and the assessment instrument has yielded promising results with respect to convergent validity and discriminant validity. The limitations of the MSCEIT included the validity of emotion perception items and the precision of emotion management items. One criticism of the MSCEIT mentioned that the way the authors determined the correct response for emotional management is problematic because the decision-making of every human being is quite different and is hugely influenced by their personality, traits, as well as cultural norms (Warwick et al., 2010).

The distinction between trait and ability measure of EI is a behavioral tendency measured through a self-report assessment. In contrast, the ability EI is the measure of one's actual ability *via* performance tests. Trait EI measure required the respondents to self-rate on a survey to predict their emotional or behavioral tendency, whereas this measure somehow could lead to inaccuracy or susceptibility to faking if respondents believe that their superior such as employer or supervisor can access their results. One of the disadvantages of self-report assessment is that the respondents can easily come across as excellent in EI skills by manipulating the results in a socially desirable or self-interest way. A further issue of self-report is that not every individual is good at judging his/her emotional abilities and tendencies, thus personal bias and misjudgment will lead to the inaccuracy of results (O'Connor et al., 2019). Unlike the self-report items, the ability test items might help to reduce respondents' personal rating bias and overlap with personality variables. Upon including the ability-based measures, the adapted SEIS not only requires test-takers to self-rate on every statement, but also requires them to answer emotional ability test that could provide a good indication of a person's ability to perceive and understand emotions. To address shortcomings of the trait measure, the current study aims to adapt the SEIS measure from trait to mixed measure of EI to enable the researchers to examine both trait and ability EI of the respondents.

### Development of the A-SEIS Measures

**Figure 1:**
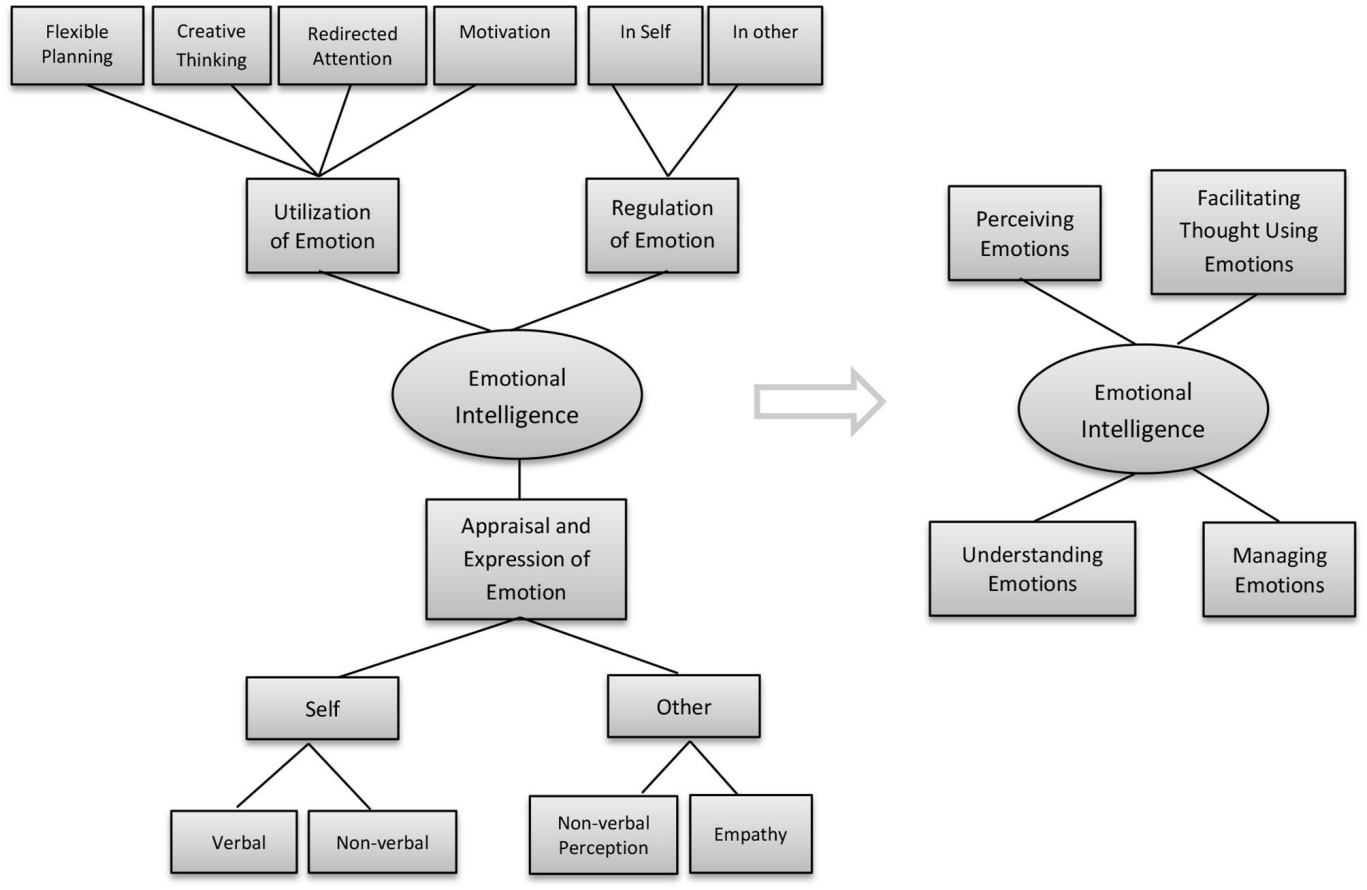
Mayer–Salovey ability-based model (1990) and the Mayer and Salovey (1997) four-branch model of emotional intelligence (EI) abilities. Reproduced from Salovey and Mayer (1990), with permission from SAGE Publications. Reproduced from Fiori and Vesely-Maillefer (2018), with permission from Springer.

As mentioned earlier, the SEIS is grounded in the older version of the ability-based model. The current study attempts to adapt the SEIS by including the construct of understand of emotions (six ability-based items) and additional 12 ability-based items into the construct of perception of emotions using the latest Mayer and Salovey (1997) four-branch ability-based model, as illustrated in Figure 1. The existing 33 trait measure items in SEIS remained in the adapted instrument. In reality, most of the EI tests were developed in western countries. EI test might indicate how people should feel, but it does not mean people will always follow such patterns of emotions. To address item validity and cultural differences in emotions, all self-report items, including understanding of emotions and perception of emotions items for the new measure were evaluated by several Malaysian experts with emotion knowledge to identify any unclear elements in the A-SEIS. Ability-based items were developed to measure the understanding of emotions through scenarios or situational tests and the perception of emotion items was evaluated *via* facial expression pictures. More specifically, the understanding of emotion construct is designed to assess the participants' ability to understand emotion through a given scenario. For example, the participants indicated which emotion of five choices was most likely to occur after witnessing a car accident. The second “perception of emotion” task assessed the participants' ability to accurately perceive emotion through facial expressions and involved several static faces such as surprise facial expressions. Participants chose one of the emotions from five alternative emotions that were best suited to the expression of the picture.

The measurement has been divided into two parts: self-report measures and ability measures. In the A-SEIS, self-report measures rely heavily on the respondents' sincerity and subjective perception of their own EI performance or skills presented by the questionnaire. On the other side, it also examines the respondents' emotional skills using a series of objective, pictures, and impersonal questions through ability measures. To summarize, the A-SEIS is going to measure an individual's ability to perceive, utilize, understand, and manage emotions based on subjective rating, situational test, and human facial expression pictures to understand how well they behave, perform, and resolve emotional-related problems in their life.

In addition, the present study also developed a new measure and scoring method for the ability-based items. In reality, it is not a single emotion that appears at a time, there could exist more than one or even mixed emotions when people encounter an emotional situation. The current study presumed that there must be one emotion best representing the emotion-related question. For the scoring methods, the researchers seek an expert evaluation on every question to figure out the best answer for each ability item and other potential emotions that might represent but not best reflect the situation. The scores of every emotion for every question can range from 5—best match emotion to 1—most irrelevant emotion, with high match emotion indicating more characteristic EI.

## Methodology

### Dimension of the A-SEIS

The research modified and developed the A-SEIS to evaluate youth EI levels using the Mayer and Salovey four-branch model of EI abilities. The adapted SEIS was used to measure the five dimensions of EI skills, including perception of emotions (22 items), understanding of emotion (6 items), managing own emotions (9 items), managing others' emotions (8 items), and utilization of emotion (6 items). The measure of EI in the instrument involved self-report measures and ability measures. The self-report items included items 1, 2, 4, 5, 12, 15, 16, 17, 18, 19, 20, 22, 24, 29, 31, 34, 35, 36, 37, 38, 39, 40, 41, 42, 43, 44, 45, 46, 47, 48, 49, 50, and 51, while the ability measures involved items 3, 6, 7, 8, 9, 10, 11, 13, 14, 21, 23, 25, 26, 27, 28, 30, 32, and 33. In sum, there will be 51 items in the instrument and total scale scores are calculated by reverse coding items 12, 46, and 51, and then summing all items. The scores can range from 51 to 255, with higher scores indicating more characteristic EI. The questionnaire responses option involved 5-point rating scale. The A-SEIS consists of self-report measures and ability measures. Items under self-report measures comprised of items. Questions' structure, answers, and content were adapted based on experts' feedbacks and advices. The draft of the A-SEIS was translated and reviewed by three experts in the field of EI and modified based on their comments to create the complete A-SEIS.

### Translation of the A-SEIS

**Figure 2:**
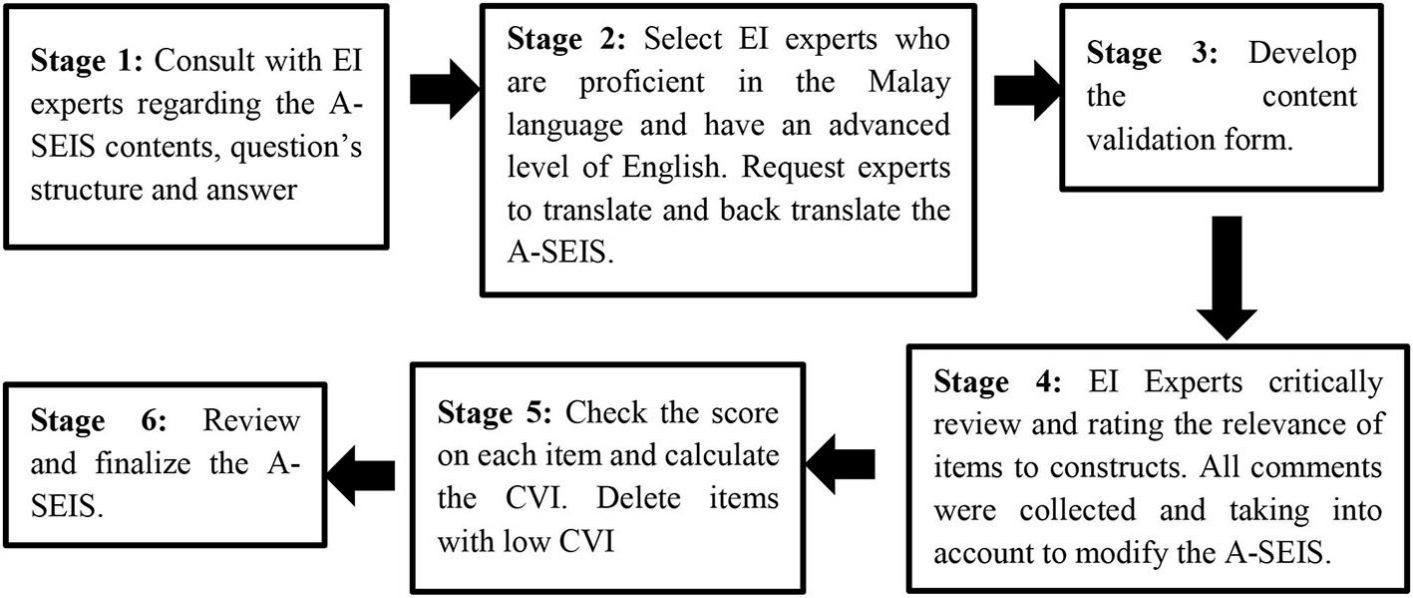
Content validation stages of the A-SEIS.

The A-SEIS was developed through the process of forward and backward translation to ensure its suitability and validity for the Malaysian population. For the forward and backward translation of the instrument, the following procedures were used (as shown in Figure 2): (1) items development and preparation, (2) translation of the A-SEIS questionnaire (forward translation), then translation of the translated Bahasa Malaysia (A-SEIS) questionnaire into the English language (back translation), (3) Prepare content validation form, (4) examine the feedbacks and rating from experts, (5) check the content validation index, (6) review and finalization of the A-SEIS.

Before adopting and translating the instrument, written permission from the main author to use and revise the SEIS was obtained. Researchers adapted the SEIS and requested three experts who are not only skillful in the domain being studied, but also proficient or specialized in the Malay language and have an advanced level of English to translate and review the contents. This was followed by the translation of the A-SEIS into the Malay language by two independent bilingual experts with a high level of language proficiency in Malay and English, who reside permanently in Malaysia and whose native language is Bahasa Melayu. Each expert generated an independent forward translation of A-SEIS from the language of English to Malay. Later, researchers checked the differences between the independent experts and decided on the final wording of the Malay version of A-SEIS.

To backward translate the Malay version A-SEIS questionnaire into the English version A-SEIS questionnaire, a Malaysian bilanguage researcher who conduct research in Ireland was recruited to translate the A-SEIS questionnaire into the English language. After completion, the researchers compared the backward translation of A-SEIS with the original SEIS questionnaire to confirm semantic equivalence and ensure that all questions are culturally appropriate and easy to understand so as to avoid any kind of inaccuracy during the translation process. Moving forward, the content validity of the instrument was evaluated to ensure that the A-SEIS is ready to use for subsequent research.

### Content Validity Index

To determine the content validity of A-SEIS, a systematic approach on validation was performed based on the evidence and the best approach. The content validity can be explained as the extent to which elements of a measurement instrument are related to and representative of the underlying variable for a particular assessment objective (Yusoff, 2019). Researchers collected evidence-based information and transforms them into potential items for inclusion in the A-SEIS. After the A-SEIS was created, the content validity of A-SEIS was examined through a review panel of experts. According to Gilbert and Prion (2016), review panels of 5–10 members are ideal for content validation purposes, the use of three experts is also acceptable, but more than 10 experts seem unnecessary for the validation process (Lynn, 1986). In the content area, each panel is supplied with the content validation form developed by the researchers. Independent of other panelists, each member was requested to evaluate the degree of relevance of each item from 1 “the item is not relevant to the measured domain” to 4 “the item is highly relevant to the measured domain.”

The CVI process is a critical stage in the adaptation and translation of the A-SEIS to ensure the overall validity and applicability of the instrument in the Malaysian context. There are two forms of CVI, namely content validity of individual items (I-CVI) and content validity of overall scale (S-CVI). As noted by Polit et al. (2007), when there are five or fewer experts, the value of the I-CVI should be 1. When more than five experts take part in the evaluation process, the standard of I-CVI can be relaxed and the value should not be <0.78. In this study, the item that meets the requirements or meets the threshold of I-CVI with a value of 1 could be considered as an evidence of good content validity, otherwise should be removed from the assessment instrument. S-CVI can be calculated by two methods: the average item levels I-CVIs (S-CVI/Ave) and universal agreement among experts on items (S-CVI/UA). The explanations of the terms are provided in Table 1.

**Table 1:** Explanation of the content validation terms.

**Term**	**Description**
CVI	Extent to which an assessment tool has an adequate or appropriate sample of items for measurement constructs
I-CVI	Proportion of experts judging the items as relevant “3” or highly relevant “4”
S-CVI	Content validity of the overall scale
S-CVI/UA	The proportion of items on an assessment instrument rated as 3 or 4 by all experts
S-CVI/Ave	The average of the I-CVI scores for all items on the scale

#### Kappa Statistic Coefficient

Kappa statistic is another important complement to CVI, which provides information about the degree of agreement beyond chance (Shrotryia and Dhanda, 2019). Although CVI is widely used to determine the content validity of the instrument, however, it does not examine possible inflated values that could affect the validity because of the possibility of chance agreement. The computation of the kappa coefficient could help researchers reduce the potential validation problems by removing any random chance agreement among experts. To obtain the kappa statistic coefficient, the calculation of the probability of chance agreement is required, that is P_C_ = [N!/A! (N -A)!] × 5^N^. In this formula, *N* = number of experts and *A* = number of panelists who agree that the item is relevant. After identifying all the I-CVI values, kappa was calculated using the formula: *K* = (I-CVI–P_C_)/(1–P_C_). If the kappa coefficient is <0.39, the score might represent a potential problematic item. If the range is within 0.40–0.59, the item is considered moderate. An item with kappa coefficient in the range of 0.60–0.74 can be rated as a good item. If the value is more than 0.74, then the item is considered excellent (Orts-Cortés et al., 2013; Zamanzadeh et al., 2015). To calculate the key in the responses, the Excel software was adopted for the study.

### Exploratory Factor Analysis

Before running the factor analysis, Kaiser–Meyer–Olkin (KMO) and Bartlett's Test of Sphericity were analyzed to examine the appropriateness of the data. According to Leech et al. (2015), the value of the KMO measure must be more than 0.70. Kaiser's criterion is one of the common methods to determine the number of factors to be extracted in an instrument. For the extraction, principal component analysis was applied to group the items into meaningful dimensionality as well as examine the relations among the observed variables.

### Confirmatory Factor Analysis

The validity testing of the instrument was performed in the framework of structural equation modeling (SEM) by using the confirmatory factor analysis (CFA) approach. The validity testing included also the partial least squares based SEM (PLS-SEM) mainly because the PLS-SEM reduces measurement errors in the structural model (Farooq et al., 2018), and also one of the constructs (Managing Own Emotions) was slightly non-normally distributed as compared to the other constructs. Thus, the researchers decided to examine the CFA through the CB-SEM approach and PLS-SEM approach, using two different software that were AMOS and SmartPLS to confirm the reliability and validity of the A-SEIS.

### Different Types of SEM: CB-SEM vs. PLS-SEM

The AMOS and SmartPLS are powerful statistical software that has been widely used by researchers to analyze SEM. Abraham et al. (2021) have discussed the purpose and applications of the CB-SEM and PLS-SEM in research. CB-SEM is more suitable to apply when the purpose of the research is theory confirmation and testing, while PLS-SEM is more suitable for theory development and predictive analysis. In the aspect of model fit indices, the PLS-SEM is still evolving. However, there are some situations where a researcher could consider the PLS-SEM instead of the CB-SEM: (1) small sample size and (2) data is not normally distributed. Dash and Paul (2021) pointed out that researchers should not view CB-SEM and PLS-SEM as competitive, while these two methods are actually complementary to each other. In general, both methods can bring valuable results in establishing and examining the structural relationship. The current study decided to utilize these two methods to have a better insight into the A-SEIS structural relationship.

## Data Analysis

### Sample Respondents

The samples in the research were randomly selected based on the cluster sampling technique from a population of research universities in Malaysia. In the present study, one university that represents the research universities was selected, namely the Universiti Putra Malaysia (UPM). Approximately, 25% of the respondents were male and 75% were female. Among the respondents, about 82.5% were Malays, 13.5% were Chinese, 0.5% (one respondent) were Indians, and 3.5% were from other races. The target respondents were chosen among the science and social science undergraduates who are currently studying in the first or second semester. For exclusion criteria, PhD, masters, second and third year UPM students were not included in the study. All respondents were recruited from the Universiti Putra Malaysia. Through fish bowl techniques, three programs that represent fields of social science were randomly selected, namely Bachelor of Education in Guidance and Counseling, Bachelor of Education in Bahasa Melayu, and Bachelor of Education in Agricultural Science. Another four programs from the fields of science were randomly chosen, namely Bachelor of Nursing, Bachelor of Nutrition and Community Health, Bachelor of Biomedical Sciences, and Bachelor of Science Environmental & Occupational Health. The researchers used intact classes in the study because it is impossible to adjust the structured academic sessions for every participant. Many researchers apply this research design in educational research due to its difficulty to perform randomization or true experimental research in educational settings (Ary et al., 1972). Recruitment of respondents was carried out during the briefing and all students were encouraged to freely volunteer themselves in this research.

The distribution of sample respondents based on fields of study is shown in Table 2. A total of 200 sample respondents were selected randomly for the research with 112 respondents representing fields of social science and 89 respondents representing fields of science. A minimum of 200 sample sizes is suggested to generate a fair representation of the population where statistic calculation could be performed for the population inference (Louangrath, 2017; as cited in Memon et al., 2020).

**Table 2:** Distribution of sample respondents in UPM.

**Categories**	***n***	**%**
**Gender**		
Male	50	25.0
Female	150	75.0
Total	200	100.0
**Races**		
Malays	165	82.5
Chinese	27	13.5
Indians	1	0.5
Others	7	3.5
Total	200	100
**Programs**		
Bachelor of Education in Guidance and Counseling	35	17.5
Bachelor of Education in Bahasa Melayu	41	20.5
Bachelor of Education in Agricultural Science	35	17.5
Bachelor of Nursing	16	8.0
Bachelor of Nutrition and Community Health	17	8.5
Bachelor of Biomedical Sciences	22	11.0
Bachelor of Science Environmental & Occupational Health	34	17.0
Total	200	100.0

### First-Phase Items Reduction: The Content Validity of A-SEIS

The present study checked the content validation and kappa coefficient of A-SEIS, which comprised 5 factors and 51 items. The analysis of the A-SEIS demonstrated that this version achieved a high CVI, where the S-CVI/Ave was 0.99 for five constructs. The instrument also achieved excellent universal agreement between the experts (S-CVI/UA = 0.98). However, item 17 did not achieve the minimum threshold because one of the experts rated the question as not relevant to the measured construct. Thus, only one item failed to achieve an I-CVI value of 1. Further analyzing, the kappa coefficient of all items was 1 except item 17, in which it was 0.472. For the reduction of the first-phase items, item 17 was removed and the researchers decided to not include this item for subsequent validity evaluation because of its poor I-CVI value. Overall, an impressive 98% (*n* = 50) of the items were evaluated as excellent and only 2% (one item) was rated as moderate. The results of the content validity of the A-SEIS are shown in Table 3.

**Table 3:** The relevance ratings on items by three experts.

	**Expert 1**	**Expert 2**	**Expert 3**		**Experts in agreement**	**I-CV1**	**UA**	**Pc**	**k**	**Result**	**Decision**
**Item**											
QI	1	1	1		3	1	1	0.125	1	Excellent	Remained
Q2	1	1	1		3	1	1	0.125	1	Excellent	Remained
Q3	1	1	1		3	1	1	0.125	1	Excellent	Remained
Q4	1	1	1		3	1	1	0.125	1	Excellent	Remained
Q5	1	1	1		3	1	1	0.125	1	Excellent	Remained
Q6	1	1	1		3	1	1	0.125	1	Excellent	Remained
Q7	1	1	1		3	1	1	0.125	1	Excellent	Remained
Q8	1	1	1		3	1	1	0.125	1	Excellent	Remained
Q9	1	1	1		3	1	1	0.125	1	Excellent	Remained
Q10	1	1	1		3	1	1	0.125	1	Excellent	Remained
Q11	1	1	1		3	1	1	0.125	1	Excellent	Remained
Q12	1	1	1		3	1	1	0.125	1	Excellent	Remained
Q13	1	1	1		3	1	1	0.125	1	Excellent	Remained
Q14	1	1	1		3	1	1	0.125	1	Excellent	Remained
Q15	1	1	1		3	1	1	0.125	1	Excellent	Remained
Q16	1	1	1		3	1	1	0.125	1	Excellent	Remained
Q17	1	0	1		2	0.67	0	0.375	0.472	Moderate	Removed
Q18	1	1	1		3	1	1	0.125	1	Excellent	Remained
Q19	1	1	1		3	1	1	0.125	1	Excellent	Remained
Q20	1	1	1		3	1	1	0.125	1	Excellent	Remained
Q21	1	1	1		3	1	1	0.125	1	Excellent	Remained
Q22	1	1	1		3	1	1	0.125	1	Excellent	Remained
Q23	1	1	1		3	1	1	0.125	1	Excellent	Remained
Q24	1	1	1		3	1	1	0.125	1	Excellent	Remained
Q25	1	1	1		3	1	1	0.125	1	Excellent	Remained
Q26	1	1	1		3	1	1	0.125	1	Excellent	Remained
Q27	1	1	1		3	1	1	0.125	1	Excellent	Remained
Q28	1	1	1		3	1	1	0.125	1	Excellent	Remained
Q29	1	1	1		3	1	1	0.125	1	Excellent	Remained
Q30	1	1	1		3	1	1	0.125	1	Excellent	Remained
Q31	1	1	1		3	1	1	0.125	1	Excellent	Remained
Q32	1	1	1		3	1	1	0.125	1	Excellent	Remained
Q33	1	1	1		3	1	1	0.125	1	Excellent	Remained
Q34	1	1	1		3	1	1	0.125	1	Excellent	Remained
Q35	1	1	1		3	1	1	0.125	1	Excellent	Remained
Q36	1	1	1		3	1	1	0.125	1	Excellent	Remained
Q37	1	1	1		3	1	1	0.125	1	Excellent	Remained
Q38	1	1	1		3	1	1	0.125	1	Excellent	Remained
Q39	1	1	1		3	1	1	0.125	1	Excellent	Remained
Q40	1	1	1		3	1	1	0.125	1	Excellent	Remained
Q41	1	1	1		3	1	1	0.125	1	Excellent	Remained
Q42	1	1	1		3	1	1	0.125	1	Excellent	Remained
Q43	1	1	1		3	1	1	0.125	1	Excellent	Remained
Q44	1	1	1		3	1	1	0.125	1	Excellent	Remained
Q45	1	1	1		3	1	1	0.125	1	Excellent	Remained
Q46	1	1	1		3	1	1	0.125	1	Excellent	Remained
Q47	1	1	1		3	1	1	0.125	1	Excellent	Remained
Q48	1	1	1		3	1	1	0.125	1	Excellent	Remained
Q49	1	1	1		3	1	1	0.125	1	Excellent	Remained
Q50	1	1	1		3	1	1	0.125	1	Excellent	Remained
Q51	1	1	1		3	1	1	0.125	1	Excellent	Remained
					**S-CVI/Ave**	0.99					
Proportion relevance	1	0.98	1	0.99	**S-CVI/UA**		0.98				

*Average Proportion of Items judged as relevant across three experts.*

### Second-Phase Items Reduction Using EFA and CFA

EFA and CFA were used for second-phase items reduction. These methods enable the researchers to investigate the inter-relationships of the items, factor structures, as well as the nature of the constructs. Several conditions were considered in the process of removal of items: (1) items with low-factor loading, (2) items that affect the internal consistency measures of the A-SEIS, and (3) items with low-average variance extracted (AVE). Before the analysis, the researchers ensured that the data were normally distributed. The total variance explained indicated that 11 factors have eigenvalues over 1 and 14 items out of 51 items showed low factor loadings and AVE. After elimination of the items, there were only 37 items remained in the A-SEIS. The analysis was performed again to re-check the factor loading, internal consistency reliability, and validity of the A-SEIS.

### Skewness, Kurtosis, Mean, Median, Mode, and Standard Deviation of the A-SEIS

After removal of the items, the researchers re-examined the skewness, kurtosis, mean, median, and mode using the SPSS software to assure the symmetry and distribution of the constructs. Tabachnick and Fidell (2007) suggested that skewness and kurtosis coefficient close to the value of 0 or <1 indicates a normal distribution data. Other methods included examining the central tendency: the median, mean, and mode as indicators of normal distribution data. For a typically symmetrical distribution, usually the value of the median and mean will fall at the same point or are equal, while the value of the mode should close to the value of the median and mean. The results in Table 4 indicated that skewness and kurtosis of the A-SEIS were <1, suggesting the entire constructs distribution falls within the normal distribution range. Furthermore, the values of the mean, median, and mode of every construct were similar, except the construct of managing own emotions showed a slight difference in the value of mean, median, and mode.

**Table 4:** Normality of the A-SEIS constructs after items deletion.

**Constructs**	**Items**	**Skewness**	**Kurtosis**	**Mean**	**Median**	**Mode**	**Std. deviation**
PER	14	−0.39	0.57	56.30	56.00	56.00	7.47
UND	6	−0.34	−0.18	17.05	17.00	16.00	2.20
MOE	8	−0.91	−0.10	25.35	27.00	28.00	3.61
MOTE	5	−0.75	0.55	30.97	31.00	31.00	4.89
UE	4	−0.49	0.12	18.80	19.00	20.00	3.35

### Correlation of the A-SEIS

Table 5 represents how five constructs of A-SEIS were correlated with one another. The coefficient correlations ranged from 0.05 to 0.49. Although most of the constructs were moderately and weakly correlated, the results showed that multicollinearity did not exist among constructs, evidence with VIF values obtained are between 1 and 10.

**Table 5:** Correlation matrix of the A-SEIS.

**A-SEIS constructs**	**PER**	**UE**	**UND**	**MOE**	**MOTE**
PER	1.00				
UE	0.33	1.00			
UND	0.13	0.14	1.00		
MOE	0.31	0.37	0.05	1.00	
MOTE	0.32	0.49	0.12	0.44	1.00

### Factor Analysis

For the third-phase analysis, the Table 6 illustrated that the KMO measures were 0.887, indicating the data is useful and appropriate for the factor analysis. While the Bartlett's Test of Sphericity was significant (*p* > 0.05), indicating the correlations among measure constructs were sufficient and non-null correlations among A-SEIS.

**Table 6:** Kaiser–Meyer–Olkin (KMO) sample adequacy test.

**KMO and Bartlett's Test**		
Kaiser–Meyer–Olkin measure of sampling adequacy		0.89
	Approx. chi-square	4,229.50
Bartlett's test of sphericity	df	666
	Sig.	0.00

**Figure 3:**
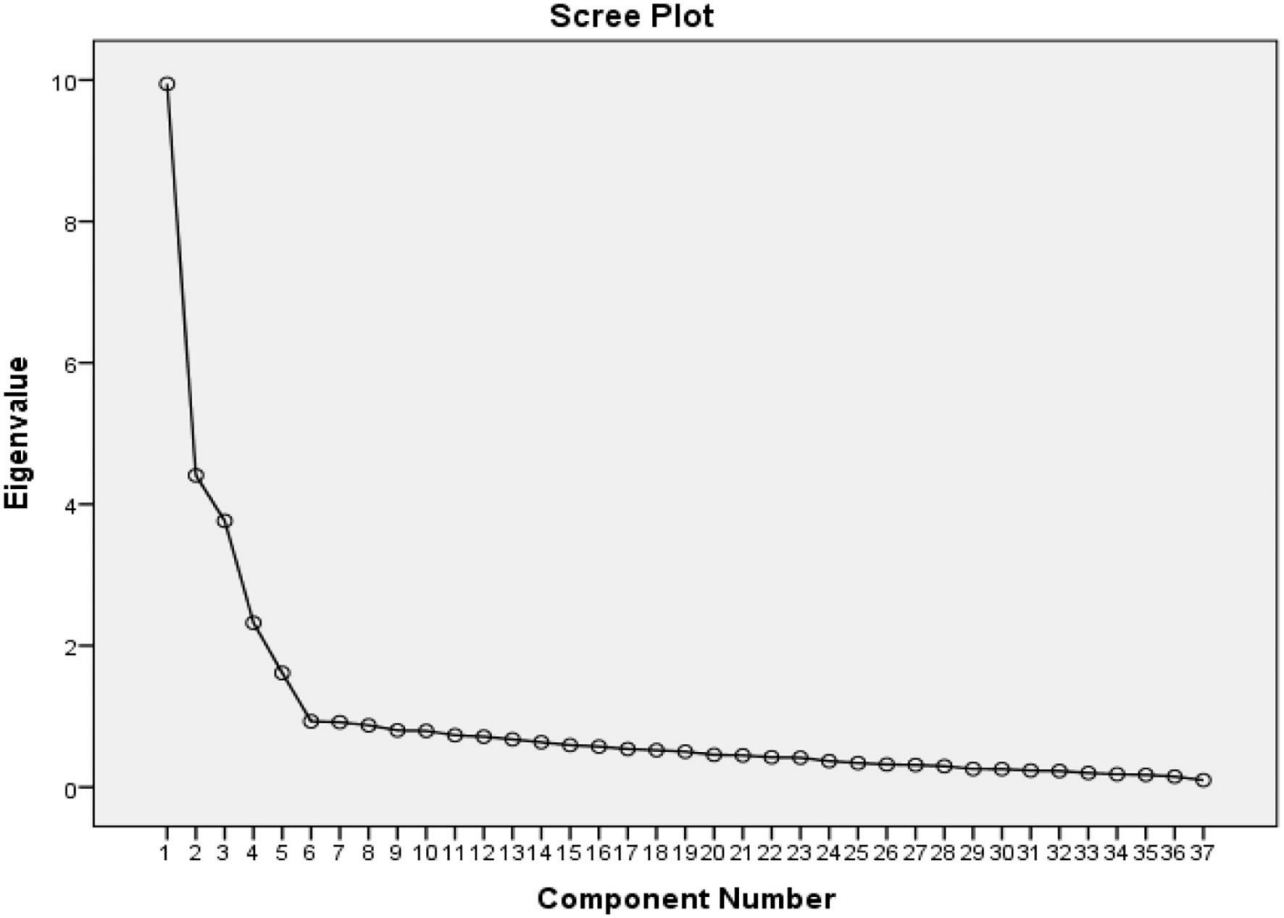
The output of Scree Plot illustrated that the A-SEIS has five factors.

In Figure 3, the PCA analysis showed that only five factors are <1 with the remaining 37 items accounting for 60% of the total variance. The first construct, perception of emotions, consisted of 14 items with an eigenvalue of 9.95. The second construct, utilization of emotions, comprised of four items with an eigenvalue of 1.61. The third construct, understanding of emotions, included six items with an eigenvalue of 3.76. The fourth construct, managing own emotions, involved eight items with an eigenvalue of 4.4. Last, the fifth construct, managing others' emotions, comprised of five items with an eigenvalue of 2.32.

Table 7 shows the items under every construct, items' factor loadings, eigenvalue, total variance explained, and the CA of each construct. As depicted, the remaining 37 items of A-SEIS were good measures of their respective construct, with factor loadings ranging from 0.57 to 0.90. CA is the criterion used to test the internal consistency of how closely each item in a construct correlated with one another. The CA of all constructs were above the 0.80, which demonstrated that the internal consistency of A-SEIS was good and well-explained by its items. Table 7 indicates the CA of 0.930 for PER, 0.807 for UE, 0.879 for UND, 0.887 for MOE, and 0.855 for MOTE. With its high CA, the researchers assumed that A-SEIS could be a good survey instrument to measure complex affective components of EI.

**Table 7:** Exploratory factor analysis on the 37 items of the A-SEIS.

**Construct &**	**Factor**	**Eigen**	**Variance**	**Cronbach's**
**item**	**loading**	**value**	**explained**	**alpha**
**C1: PER**		9.95	26.88	0.930
Q6	0.85			
Q7	0.74			
Q8	0.63			
Q9	0.67			
Q12	0.78			
Q28	0.69			
Q31	0.65			
Q36	0.80			
Q37	0.57			
Q40	0.59			
Q43	0.73			
Q47	0.64			
Q50	0.90			
Q51	0.69			
**C2:UE**		1.61	4.36	0.807
Q15	0.76			
Q16	0.76			
Q35	0.61			
Q38	0.79			
**C3: UND**		3.76	10.17	0.879
Q3	0.81			
Q13	0.76			
Q14	0.71			
Q30	0.89			
Q32	0.67			
Q33	0.87			
**C4: MOE**		4.41	11.92	0.887
Q2	0.76			
Q4	0.69			
Q19	0.72			
Q22	0.81			
Q39	0.71			
Q41	0.59			
Q46	0.74			
Q49	0.73			
**C5: MOTE**		2.32	6.28	0.855
Q1	0.72			
Q5	0.70			
Q24	0.85			
Q34	0.70			
Q44	0.73			

### Confirmatory Factor Analysis

#### CB-SEM

**Figure 4:**
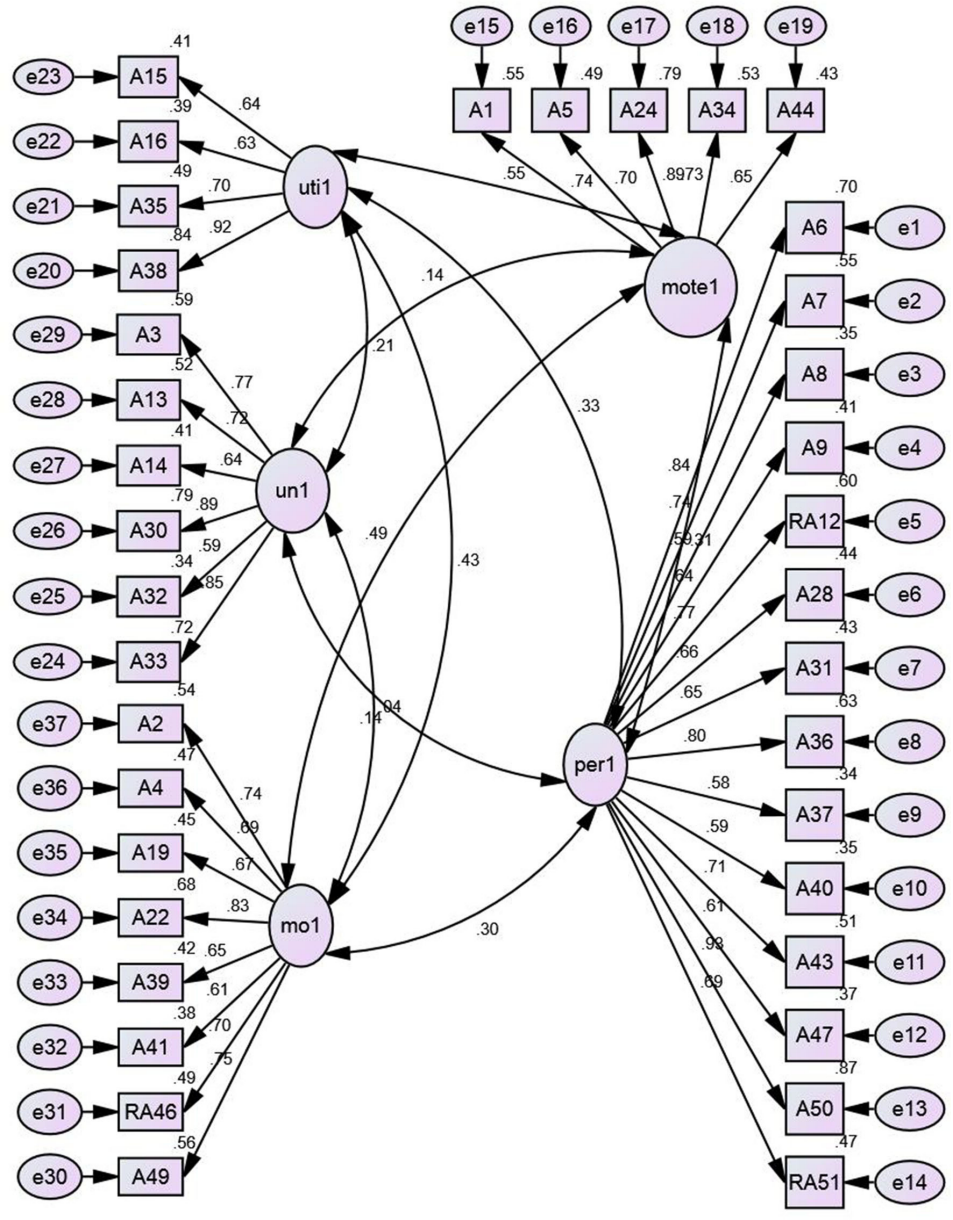
Model diagram of A-SEIS.

The Figure 4 presented the constructs and model fit of A-SEIS was analyzed using the analysis of moment structure (AMOS) software. The results indicated that model fit was fulfilled because all of the fit indices had met the minimum requirements: Chi-square/degree of freedom (χ^2^/*df* ) = 1.41, *p* = 0.00, comparative fit index (CFI) = 0.934, the Tucker–Lewis fit index (TLI) = 0.929, and root mean square of error approximation (RMSEA) = 0.045. At least three to four fit indices are required to confirm the model fit. Usually, the researchers can affirm that their measurements have a good model fit when the χ^2^/*df* is < 5, CFI is more than 0.9, TLI is more than 0.9, and RMSEA is <0.08 (as cited from the Hadie et al., 2017). In the study, all constructs were distinct from each other because the correlation coefficients between the constructs were not more than 0.85. Table 8 presents the unstandardized and standardized parameter estimates of the A-SEIS. All items are statistically significant with a *p*-value of 0.001. The squared multiple correlations (*R*^2^) explained the amount of items' variance explained by the respective constructs.

**Table 8:** Findings of CB-SEM using the AMOS.

**Construct &**	**Unstandardized**	**Standardized**	***R^**2**^***	**S.E**	**C.R**	***P***
**item**	**estimation**	**estimation**				
**C1: PER**						
Q6	1.000	0.836	0.70			
Q7	0.827	0.744	0.55	0.067	12.314	***
Q8	0.751	0.594	0.35	0.082	9.125	***
Q9	0.814	0.640	0.41	0.081	10.033	***
Q12	0.924	0.775	0.60	0.071	13.063	***
Q28	0.831	0.662	0.44	0.079	10.484	***
Q31	0.896	0.655	0.43	0.087	10.325	***
Q36	0.955	0.796	0.63	0.070	13.626	***
Q37	0.723	0.583	0.34	0.081	8.912	***
Q40	0.696	0.593	0.35	0.076	9.098	***
Q43	0.846	0.713	0.51	0.073	11.578	***
Q47	0.800	0.606	0.37	0.086	9.347	***
Q50	1.098	0.931	0.87	0.062	17.712	***
Q51	0.905	0.685	0.47	0.082	10.975	***
**C2:UE**						
Q38	1.000	0.919	0.84			
Q35	0.787	0.701	0.49	0.074	10.655	***
Q16	0.686	0.625	0.39	0.074	9.276	***
Q15	0.692	0.642	0.41	0.072	9.587	***
**C3: UND**						
Q33	1.000	0.851	0.72			
Q32	0.701	0.586	0.34	0.080	8.773	***
Q30	1.065	0.887	0.79	0.069	15.542	***
Q14	0.730	0.642	0.41	0.074	9.847	***
Q13	0.881	0.722	0.52	0.076	11.54	***
Q3	0.904	0.767	0.59	0.072	12.598	***
**C4: MOE**						
Q49	1	0.746	0.56			
Q46	1.009	0.701	0.49	0.104	9.728	***
Q41	0.746	0.612	0.38	0.089	8.429	***
Q39	0.916	0.651	0.42	0.102	8.995	***
Q22	1.262	0.827	0.69	0.109	11.600	***
Q19	1.012	0.673	0.45	0.109	9.316	***
Q4	0.997	0.689	0.47	0.104	9.546	***
Q2	1.103	0.736	0.54	0.108	10.244	***
**C5: MOTE**						
Q1	1	0.742	0.55			
Q5	0.735	0.699	0.49	0.077	9.584	***
Q24	1.176	0.886	0.79	0.098	12.036	***
Q34	0.809	0.731	0.53	0.080	10.048	***
Q44	0.854	0.652	0.43	0.096	8.918	***

#### PLS-SEM

**Figure 5:**
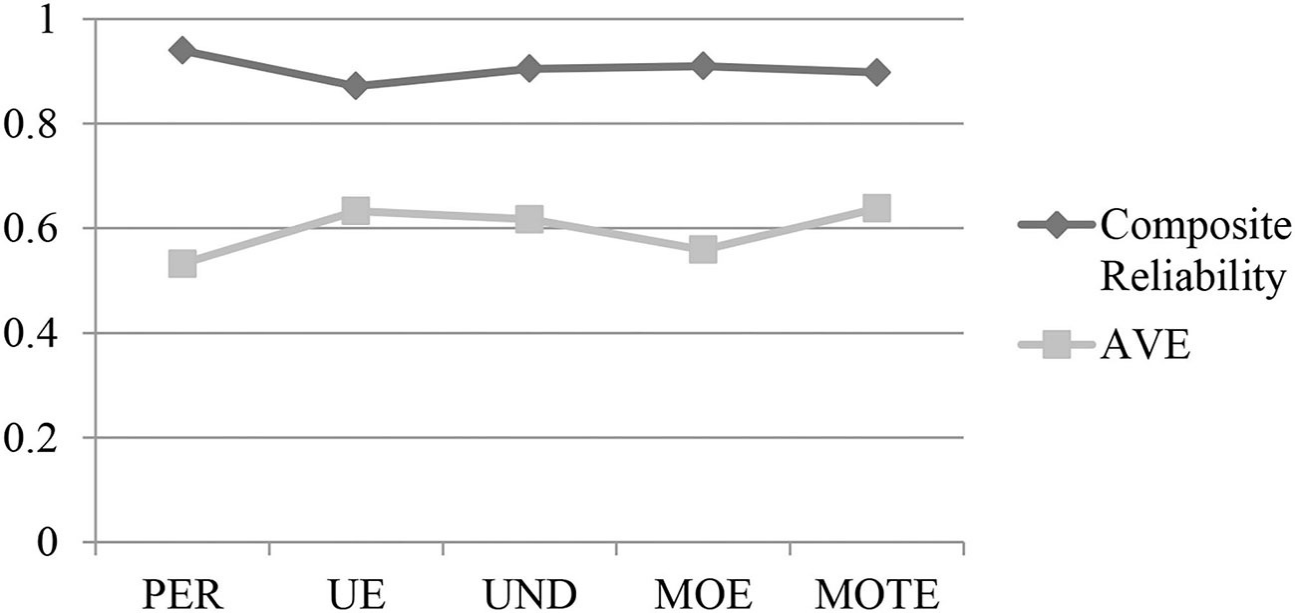
Composite reliability and convergent validity of EI constructs.

A replication of CFA was performed based on the PLS-SEM approach using the smartPLS. The composite reliability, CA, and AVE were being examined to measure the internal consistency reliability of adapted SEIS. As shown in Figure 5, the results of composite reliability demonstrated that all five constructs were above the minimum critical value (0.70) as suggested by Cohen (1988). Table 9 reveals the composite reliability value appeared to be 0.940 (PER), 0.872 (UE), 0.905 (UND), 0.910 (MOE), and 0.898 (MOTE).

**Table 9:** The internal consistency reliability of the adapted SEIS based on the constructs of PER, UE, UND, MOE, and MOTE.

**Construct**	**Composite**	**Average variance**
	**reliability**	**extracted**
PER	0.940	0.532
UE	0.872	0.633
UND	0.905	0.617
MOE	0.910	0.559
MOTE	0.898	0.638

The AVE value of all constructs should achieve at least a minimum threshold of 0.50 as suggested by Hair et al. (2017). The results presented that PER (0.532) was beyond the required lowest threshold value of 0.50. This was also applicable to UE (0.633), UND (0.617), MOE (0.559), and MOTE (0.638). By referring to Figure 5, all the lines present that these five reflective constructs have achieved the minimum threshold value, and therefore, the instruments used in measuring the five constructs have established a high level of convergent validity. To summarize, the present study concluded that the A-SEIS has met a great degree of internal consistency, reliability, and validity. The complete results are presented in Table 9.

#### Discriminant Validity of the Adapted SEIS

The Fornell–Larcker criterion is one of the general methods to evaluate the discriminant validity. The AVE must be checked if the researchers applied the Fornell–Larcker criterion in their study. Table 10 shows the results of the Fornell–Larcker criterion assessment with the reflective construct MOE has a value of 0.748 for the square root of its AVE. The value is higher than the MOTE (0.454), PER (0.316), UE (0.396), and UND (0.056). As for the reflective construct of MOTE, it has a value of 0.799 for the square root of its AVE which is greater than PER (0.344), UE (0.511), and UND (0.141). The square root of AVE for PER is 0.729 which is higher than UE (0.340) and UND (0.137). For UE, the value of the square root of its AVE is 0.796, which is greater than UND (0.154). Last, the reflective construct for UND has a value of 0.785 for the square root of its AVE.

**Table 10:** Fornell–Larcker criterion for the constructs PER, UE, UND, MOE, and MOTE.

	**MOE**	**MOTE**	**PER**	**UE**	**UND**
MOE	0.748				
MOTE	0.454	0.799			
PER	0.316	0.344	0.729		
UE	0.396	0.511	0.340	0.796	
UND	0.056	0.141	0.137	0.154	0.785

**Figure 6:**
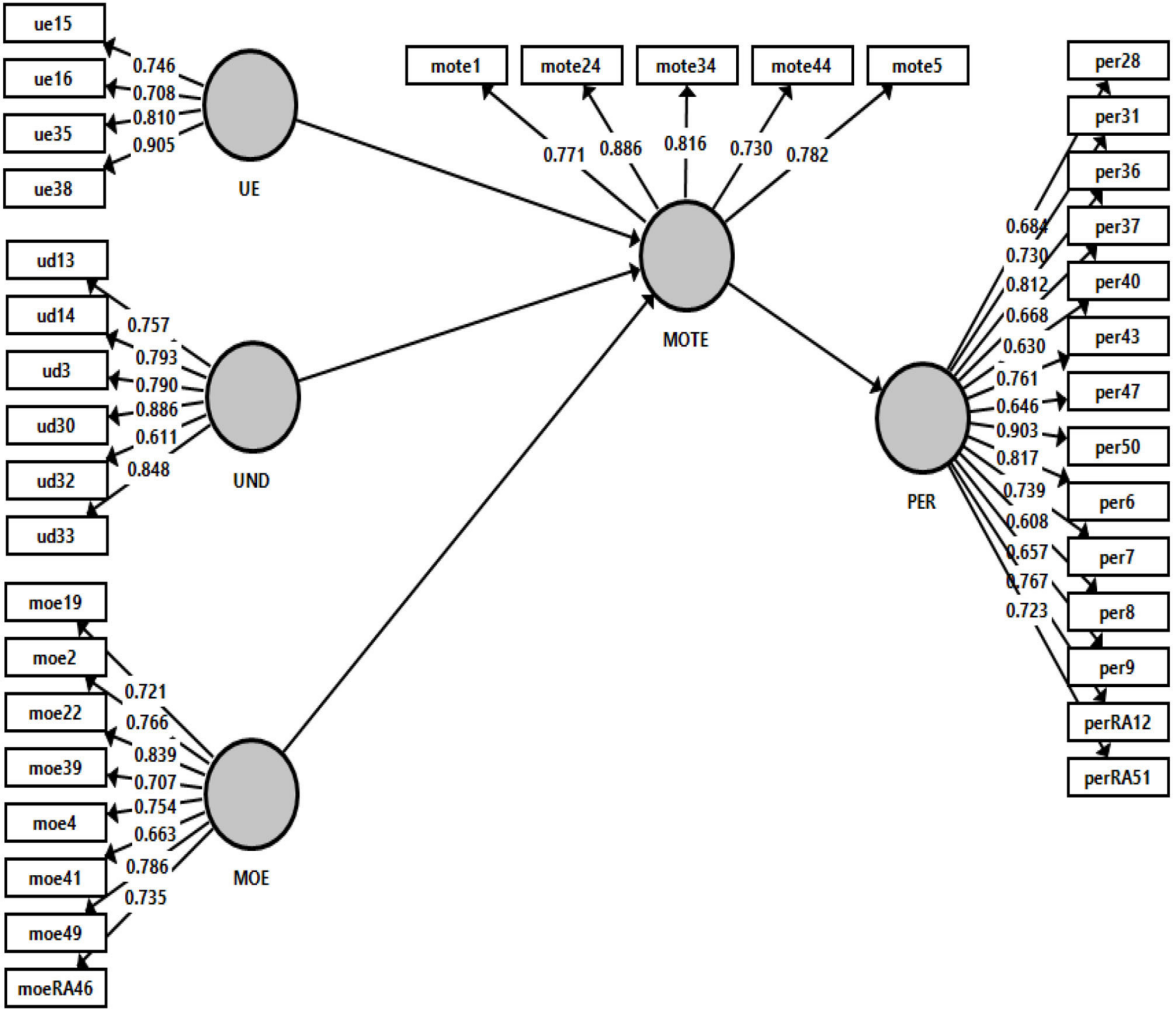
Outer loading for the measurement model.

Table 11 and Figure 6 presented the items' cross-loading that are reflected on the five different latent constructs (i.e., PER, UE, UND, MOE, and MOTE). As stated by Wong (2019), the outer loadings of every item must be examined to ensure the associated items are captured by the latent constructs. With this, the outer loadings of the item must exceed a threshold level 0.4. The results indicated that items moe19, moe2, moe22, moe4, moe49, and moeRA46 load high on its corresponding construct MOE and much lower on other constructs MOTE, PER, UE, and UND. Items mote1, mote24, mote34, mote44, and mote5 also load high on its corresponding construct MOTE but lower on other constructs MOE, PER, UE, and UND. Items per31, per36, per43, per50, per6, per7, perRA12, and perRA15 also appeared to load high on its corresponding construct PER but much lower on other constructs UE, UND, MOE, and MOTE. Furthermore, items ue15, ue16, ue35, and ue38 seem to load high on their corresponding constructs UE and low on other constructs PER, UND, MOE, and MOTE. Last, items ud13, ud14, ud3, ud30, and ud33 load high on their corresponding construct UND and much lower on other constructs PER, UE, MOE, and MOTE. The findings of the study showed that discriminant validity has been established for all five constructs as the cross-loadings of items were loaded high on the respective constructs. Chan and Yoon (2018) mentioned that indicators with outer loadings values more than the threshold value of 0.708 should remain in the instrument. In Figure 6, it is illustrated that most of the items that remained in the measurement model have outer loadings values beyond the threshold value of 0.708. Although some items (moe39, moe41, per28, per37, per40, per47, per8, per9, and ud32) did not achieve the minimum threshold value; however, these items were not removed because their AVE values are still acceptable with the inclusion of the items.

**Table 11:** Cross loadings for the constructs PER, UE, UND, MOE, and MOTE.

	**MOE**	**MOTE**	**PER**	**UE**	**UND**
moe19	0.721	0.276	0.223	0.240	0.019
moe2	0.766	0.320	0.262	0.199	−0.038
moe22	0.839	0.360	0.242	0.362	0.010
moe39	0.707	0.310	0.251	0.180	0.022
moe4	0.754	0.411	0.243	0.250	0.061
moe41	0.663	0.326	0.242	0.425	0.054
moe49	0.786	0.389	0.192	0.381	0.086
moeRA46	0.735	0.277	0.249	0.313	0.117
mote1	0.402	0.771	0.257	0.325	0.108
mote24	0.378	0.886	0.255	0.437	0.151
mote34	0.409	0.816	0.330	0.469	0.124
mote44	0.248	0.730	0.299	0.373	0.090
mote5	0.359	0.782	0.229	0.421	0.087
per28	0.140	0.180	0.684	0.187	0.104
per31	0.271	0.361	0.730	0.283	0.135
per36	0.240	0.257	0.812	0.303	0.082
per37	0.289	0.337	0.668	0.311	0.191
per40	0.339	0.180	0.630	0.281	0.090
per43	0.276	0.280	0.761	0.216	0.090
per47	0.224	0.243	0.646	0.145	0.080
per50	0.252	0.284	0.903	0.333	0.134
per6	0.179	0.169	0.817	0.211	0.045
per7	0.162	0.228	0.739	0.244	0.120
per8	0.110	0.155	0.608	0.169	0.114
per9	0.206	0.160	0.657	0.162	0.004
perRA12	0.148	0.195	0.767	0.214	0.065
perRA51	0.262	0.255	0.723	0.276	0.041
ud13	0.031	0.101	0.115	0.148	0.757
ud14	0.067	0.162	0.070	0.107	0.793
ud3	0.080	0.083	0.209	0.115	0.790
ud30	0.034	0.128	0.123	0.165	0.886
ud32	0.024	0.051	0.049	0.047	0.611
ud33	−0.001	0.061	0.089	0.107	0.848
ue15	0.214	0.339	0.332	0.746	0.094
ue16	0.225	0.289	0.176	0.708	0.047
ue35	0.406	0.475	0.299	0.810	0.119
ue38	0.365	0.475	0.266	0.905	0.199

**Figure 7:**
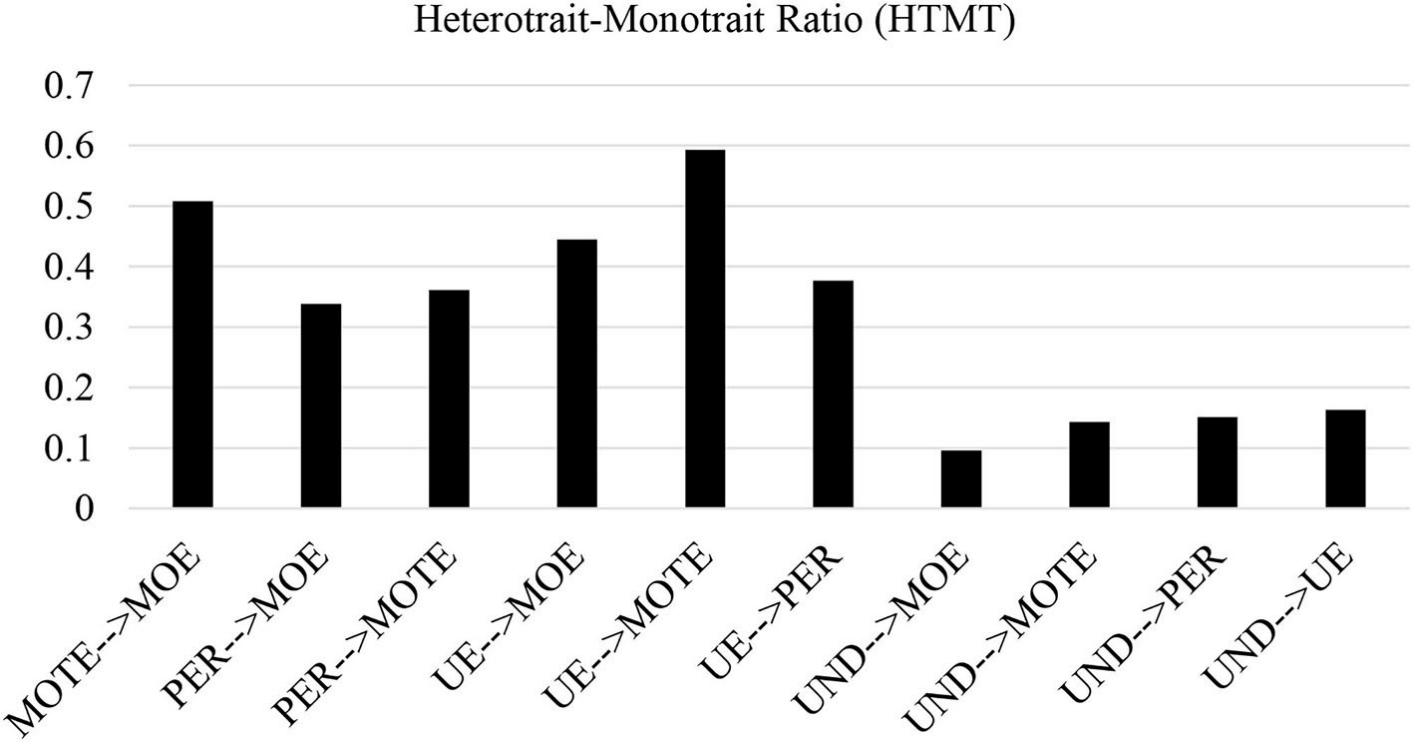
Heterotrait–monotrait ratio (HTMT).

The heterotrait–monotrait ratio (HTMT) is presented in Table 12 and bar charts in Figure 7. Henseler et al. (2015) proposed the HTMT as an alternative method that could reliably detect the discriminant validity based on the multitrait–multimethod matrix (as cited in Wong, 2019). The results revealed that the A-SEIS constructs in the proposed path model are conceptually distinct as the constructs fall under the maximum threshold value of 0.85.

**Table 12:** Heterotrait–monotrait ratio (HTMT) for the constructs PER, UE, UND, MOE, and MOTE.

	**MOE**	**MOTE**	**PER**	**UE**	**UND**
MOE					
MOTE	0.508				
PER	0.338	0.361			
UE	0.445	0.593	0.377		
UND	0.096	0.143	0.151	0.163	

Moreover, the bar charts shown in Figure 7 also clearly indicate the discriminant validity has been established. The bar charts illustrated the HTMT for MOTE à MOE was 0.508, PER à MOE was 0.338, PER à MOTE was 0.361, UE à MOE was 0.445, UE à MOTE was 0.593, UE à PER was 0.377, UND à MOE was 0.096, UND à MOTE was 0.143, UND à PER was 0.151, and UND à UE was 0.163.

## Discussion

The objectives of the study were to adapt and translate the SEIS instrument as well as examine its reliability and validity to use in the Malaysian context. The findings proved that the content validity, reliability, and validity of the A-SEIS were excellent. Three experts' ratings on CVI indicated that there must be a 100% agreement on each item. The results of CVI and kappa coefficient were excellent and only one item has been removed from the A-SEIS. Based on the results of EFA, the instrument achieved five-factor structures and the measurement dimensions were aligned with the concept of the model proposed by the Mayer Salovey four branches of EI. Through the exploratory factor analysis (EFA), CB-SEM, and PLS-SEM analysis, the researchers concluded that the reliability, convergent validity, and discriminant validity of the A-SEIS were good and excess the minimum level of threshold. The findings evidenced that the assessment is suitable to apply in the Malaysian context. The results of the heterotrait–monotrait ratio (HTMT) also indicated that the discriminant validity of the instrument is well-established in the study as all the latent constructs were conceptually distinct from each other.

**Figure 8:**
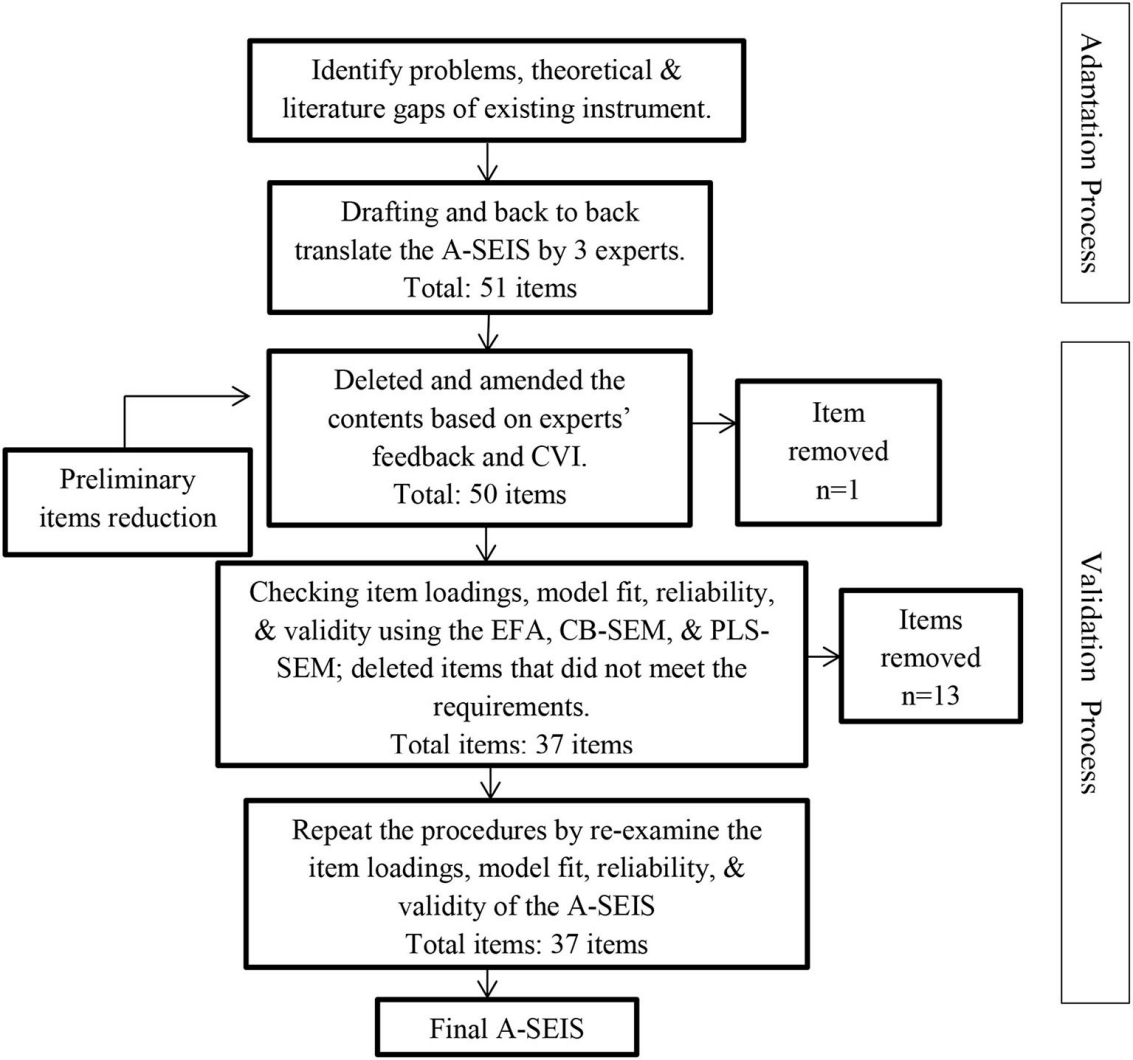
Items reduction procedures of A-SEIS.

In Figure 8, it showed that the adapted English and Malay Version of the A-SEIS had the A-SEIS had a five-factor structure comprised of 37 items with 14 items for the perception of emotions, 4 items for the utilization of emotions, 6 items for the understanding of emotions, 8 items for managing own emotions, and 5 items for managing others' emotions. Despite the current study shedding light on the adapted EI instrument' reliability and validity to apply in the Malaysian context, it has several limitations. First, the samples were selected among young adults with ages ranging from 20 to 22 years old who are studying at UPM. The second limitation is the studies on A-SEIS are relatively new, thus further investigation is especially needed on its applications in young adults. For future research, the researchers recommended an investigation of the influence of age, study field, and gender on the results of the A-SEIS. In addition, it is advisable to re-examine the content validity of the A-SEIS in each country because the norms and interpretation of emotions are very broad and might be culturally different. Last, the study highly recommended the EI researchers to expand the implication of the A-SEIS among the young population in Malaysia as well as in non-Western cultures.

## Conclusion

Based on the results of content validity, EFA, CB-SEM statistical analysis, and PLS-SEM statistical analysis, the researchers concluded that the A-SEIS is a reliable and valid measurement instrument that can be used to examine Malaysian young adults' EI.

## Data Availability Statement

The raw data supporting the conclusions of this article will be made available by the authors, without undue reservation.

## Ethics Statement

The studies involving human participants were reviewed and approved by Unit of Ethics Research, Universiti Putra Malaysia. The patients/participants provided their written informed consent to participate in this study.

## Author Contributions

NAA and SMP contributed to the adaptation of the instrument and design of the study. KST performed the statistical analysis and discussion. SMP searched for literature and re-check the final data. NAA and KST wrote the manuscript. All authors contributed to manuscript revision, discussion, and approved the submitted version.

## Funding

The authors wish to express their deepest gratitude to the *Geran Insentif Penyelidikan untuk Pengajaran dan Pembelajaran* (GIPP 9323769) Centre for Academic Development (CADe), and the Faculty of Educational Studies, Universiti Putra Malaysia for funding this article.

## Conflict of Interest

The authors declare that the research was conducted in the absence of any commercial or financial relationships that could be construed as a potential conflict of interest.

## Publisher's Note

All claims expressed in this article are solely those of the authors and do not necessarily represent those of their affiliated organizations, or those of the publisher, the editors and the reviewers. Any product that may be evaluated in this article, or claim that may be made by its manufacturer, is not guaranteed or endorsed by the publisher.
